# Clinical efficacy and imaging evaluation of recombinant tissue plasminogen activator thrombolytic therapy in patients with wake up stroke: A randomized controlled trial

**DOI:** 10.1097/MD.0000000000021958

**Published:** 2020-09-04

**Authors:** Chun-Yang Zhang, Bin Yang, Dong-Mei Li, Qiu-Yan Shi, Hong Li, Yan-Ling Li, Cui-Lan Wang

**Affiliations:** North China University of Science and Technology Affilated Hospital, Hebei, China.

**Keywords:** clinical trials, protocol, recombinant tissue plasminogen activator, wake up stroke

## Abstract

**Introduction:**

Wake up stroke starts in sleep and is a more common form of ischemic stroke. At present, it is still controversial whether wake up stroke can be treated with thrombolytic therapy. Therefore, this study will combine imaging techniques to assess the onset time of wake up stroke patients, and to analyze the imaging characteristics of wake up stroke patients and patients suitable for thrombolytic therapy within the time window.

**Methods/design:**

This study will be a single-blinded, randomized controlled trial with 2 parallel groups. It will be conducted at North China University of science and technology affiliated hospital.

**Discussion:**

There is no consistent conclusion about the pathogenesis of wake up stroke. Wake up stroke is more likely to manifest as small vessel disease. The incidence of wake up stroke patients is relatively high, and the effectiveness and safety of intravenous thrombolysis under the guidance of multimode imaging therapy in wake up stroke need to be further explored by prospective, large-scale studies.

**Trial registration:**

ClinicalTrials.gov, ChiCTR2000034402, Registered on 05 July 2020

## 1 Introduction

The patient was normal when he fell asleep at night, but after waking up, he developed stroke symptoms.^[1]^ This phenomenon was defined as wake up stroke (WUS). Due to the inability to accurately determine the actual time of onset of this part of patients, according to the criteria of stroke diagnosis and treatment guidelines, this part of patients are often excluded from recombinant tissue plasminogen activator (rt-PA) treatment. WUS is relatively common clinically, accounting for about one-quarter of patients with new-onset ischemic stroke,^[2]^ but the statistical data obtained by different studies are quite different. A population-based study in the United States in 2005 found that^[3]^ nearly 58,000 WUS patients in the United States were seen in the emergency department in 2005. The CASPR report^[4]^ the data of 11 hospitals in 5 different areas of California surveyed by it showed that the proportion of WUS accounted for about 8% of ischemic stroke, other states such as Houston is about 6.4%, Boston is about 27%.^[5,6]^ A 4-year study in the UK found that the incidence of WUS is about 25% (135/545).^[7]^ The stroke registration from Canada found that WUS accounted for 13.5%.^[8]^ Lausanne, Switzerland, reported that the incidence of WUS was as high as 33.1%.^[9]^ In the Asian region, Japan and South Korea accounted for 9.7% and 27.8% of WUS occurrences.^[10,11]^ China currently lacks epidemiological surveys on WUS. Although it is currently difficult to draw a uniform ratio from the existing statistical data, we can find that WUS is more common clinically. Data from clinical and imaging studies indicate that the onset time of most patients with ischemic stroke is between 6:00 and 12:00 in the morning,^[12]^ showing a peak in the morning. Therefore, scholars speculate that there may be a considerable proportion of WUS patients whose onset time is close to awakening, and there is still the possibility of thrombolytic therapy. In recent years, multimodal imaging techniques have been used to evaluate the ischemic penumbra after cerebral ischemia in WUS patients, thereby guiding thrombolytic therapy.^[13]^ There have been some clinical studies based on multimodal imaging techniques to guide WUS thrombolytic therapy and found that a considerable portion of WUS patients can benefit from thrombolysis,^[14]^ but most of these studies are relatively small samples and are observational studies. The use of rt-PA thrombolytic therapy in the early stage is one of the most effective treatment methods for acute cerebral infarction, but due to the limitation of time window, the proportion of patients benefiting is not high. Although the European Acute Stroke Collaboration Study extended the time window for intravenous thrombolysis, the proportion of thrombolysis in China is still <2%.^[15]^ Whether WUS can be used for thrombolytic therapy is still controversial. Therefore, this study will combine the imaging technology to assess the onset time of WUS patients, and analyze the imaging characteristics of WUS patients and patients within the time window suitable for thrombolytic therapy.^[16]^ This study aimed to observe the efficacy of intravenous thrombolysis in WUS patients.

## 2 Methods/design

### 2.1 Study design and settings

**Figure 1:**
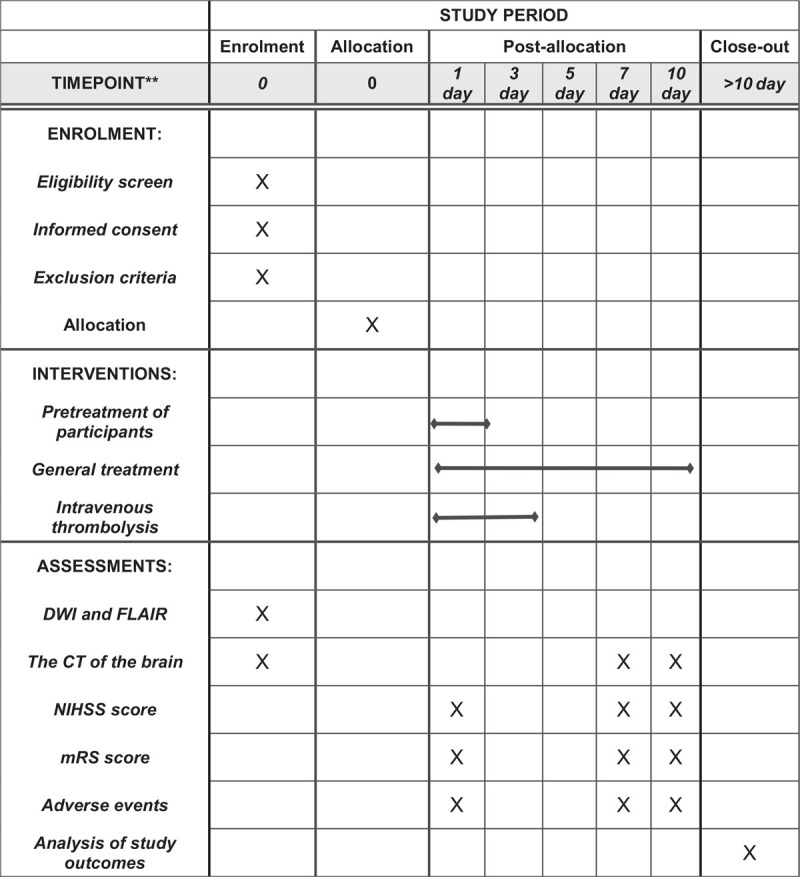
SPIRIT figure for the schedule of enrollment, interventions, and assessments. mRS = modified RanKin scale, NIHSS = National Institutes of Health Stroke Scale, SPIRIT = Standard Protocol Items: Recommendations for Interventional Trials.

This study will be a single-blinded, randomized controlled trial with 2 parallel groups. It will be conducted at North China University of Science and Technology-affiliated hospital. This protocol was written and based on Standard Protocol Items: Recommendations for Interventional Trials guidelines. If they agree, they will sign an informed consent form. Only those participants who read and agree to the protocol and who sign the informed consent form will take part of the study, following the schedule described in Figure 1.

### 2.2 Ethical

Before this clinical trial begins, we will submit the research protocol to the ethics committee for review. We will not recruit participants until the ethics committee agrees. This study will be approved by the Ethics Committee of North China University of Science and Technology-affiliated hospital. It will be conducted in accordance with the protocol. The rules of confidentiality will be respected.

### 2.3 Patients participating in the trial

#### 2.3.1 Participants

We will continuously include patients with ischemic stroke who are hospitalized in the Affiliated Hospital of North China University of Technology. We will refer to the diagnostic criteria for ischemic stroke developed by the Neurological Branch of the Chinese Medical Association.

#### 2.3.2 Inclusion criteria

1. Time of onset: last appeared to be normal until symptoms will be found <12 hours and >4.5 hours;
2. The patient was found to be awake or witnesses found neurological deficits;
3. NIHSS score ≥4 points;
4. No changes or early changes are insufficient to the one-third distribution area of the middle cerebral artery;
5. DWI/FLAIR imaging performance mismatch (DWI imaging and FLAIR imaging);
6. All other indications for intravenous thrombolysis;
7. Patients with large vessel occlusion are treated with endovascular treatment. The treatment principles refer to “Chinese Expert Consensus on Intravascular Treatment of Acute Ischemic Stroke.”

#### 2.3.3 Exclusion criteria

Patients will be excluded if they meet the following criteria:

Severe patients with malignant hypertension, severe arrhythmia, and acute myocardial infarction;
Patients with recent acute infection, trauma, surgery, diabetic ketoacidosis, and hyperosmolar coma;
Liver patients with severe primary diseases such as kidney, hematopoietic system, and so on;
Patients with tumor and other organ failures.

One of the above is excluded.

### 2.4 Pretreatment of participants

#### 2.4.1 Collection of clinical data

All hospitalized patients with ischemic stroke have a special clinician responsible for collecting general information on admission. General information collected includes name, age, sex, weight, home address, contact person, contact phone number, previous history of hypertension, diabetes, coronary heart disease, atrial fibrillation, hyperlipidemia, smoking history, drinking history, and so on. Factors, as well as the patient's medication history, epilepsy, migraine, trauma, infection, pregnancy history and imaging data.

#### 2.4.2 Grouping method

We will be divided into awakening stroke group (wake group whose morbidity is not exact) and ischemic stroke group (the group clarifying the time of onset) according to whether the onset time of the patient is clear. The awakening group is divided into the awakening group with thrombolytic therapy and the awakening group without thrombolysis according to whether they receive thrombolytic therapy.

#### 2.4.3 Laboratory examination

For patients who meet the inclusion criteria, blood routine (white blood cells, hemoglobin, platelets, etc.), biochemistry (liver function, kidney function, myocardial enzymes, blood lipids, and so on), blood coagulation (activated partial thromboplastin time, blood coagulation) time, plasma prothrombin time, and so on), conventional electrocardiogram, chest radiograph and other examinations.

### 2.5 Intervention method

#### 2.5.1 General treatment

1. Oxygen inhalation: Patients without hypoxemia do not need routine oxygen inhalation. Monitoring of oxygen saturation <94% or blood gas analysis suggesting oxygen insufflation when hypoxia is indicated. Patients with severe respiratory tract dysfunction are given airway support (tracheal intubation or incision) and ventilator-assisted ventilation.
2. Cardiac function monitoring: Routine electrocardiogram examination should be performed within 24 hours after acute ischemic stroke, and continuous multifunctional electrocardiogram monitor should be given for monitoring if necessary. Drugs that increase the burden on the heart should be avoided.
3. Body temperature management: For patients with elevated body temperature, the cause of fever should be clarified positively. If there is infection, antibiotic treatment is given, and patients with body temperature >38°C should be given cooling treatment.
4. Blood pressure control: Patients with ischemic stroke have elevated blood pressure in the acute phase and generally do not need special treatment. In most patients, blood pressure will drop by itself within 24 hours of onset. If the patient's systolic blood pressure ≥200 mmHg and/or diastolic blood pressure ≥110 mmHg after hospital admission, antihypertensive treatment should be given. Avoid reducing blood pressure too quickly to avoid affecting cerebral perfusion. The blood pressure of thrombolytic patients should be controlled at systolic blood pressure <180 mmHg, diastolic blood pressure <100 mmHg, and blood pressure changes should be monitored dynamically within 24 hours after thrombolysis. If persistent hypotension occurs after stroke, we will actively look for the cause and give treatment such as fluid replacement and boosting.
5. Blood glucose: When blood glucose exceeds 10 mmol/L, insulin hypoglycemic therapy is given. We will adjust the amount of insulin depending on the blood glucose level. We will regularly monitor blood sugar, and control blood sugar to 7.7 ∼ 10mmol/L. When the blood glucose is <3.3 mmol/L, we will give 10% to 20% glucose oral or injection treatment to maintain the blood glucose at a normal level.
6. Nutritional support: Patients who can eat normally do not need additional supplemental nutrition. For those patients with unconsciousness or inability to eat by mouth, we will talk about indwelling gastric tube, nasal feeding liquid food, or enteral nutrition supplements.

#### 2.5.2 Treatment solutions

Patients in the defined time-onset group will be given intravenous thrombolysis. The awakening group performs multimodal magnetic resonance imaging (MRI) screening (DWI and FLAIR) on the basis of meeting the pre-set conditions. If the MRI indicates that the DWI-FLAIR does not match, intravenous thrombolytic therapy will be given; if the MRI shows DWI-FLAIR matching, it will be given general treatment. Intravenous thrombolysis uses rt-PA. One-time dosage is 0.9 mg/kg, and the maximum dose does not exceed 90 mg. First, a 10% dose of intravenous bolus was given, and the rest of the dose continued to be infused intravenously for a total of 60 minutes. Thrombolysis is completed in the intensive care unit of the neurology department. During the thrombolysis process, the patient's consciousness and changes in limb muscle strength are closely observed. During this period, we will monitor patients’ vital signs, blood oxygen saturation, and give them treatments such as free radical scavenging and stable plaque. Recheck the computed tomography (CT) of the brain after 24 hours. If no intracranial hemorrhage is found, antiplatelet aggregation treatment (0.1 g oral aspirin tablets) should be given, and acupuncture and rehabilitation should be given after the condition is stable. Patients in the WUS group without thrombolysis received aspirin 300 mg orally as soon as possible after admission, and then changed to 100 mg/day. Other treatment methods will be the same as those of thrombolytic therapy.

### 2.6 Outcome measures

#### 2.6.1 Safety evaluation after thrombolysis

The occurrence time, treatment measures, and results of various adverse events such as intracranial hemorrhage, skin, gums, and digestive tract in patients undergoing thrombolytic therapy are recorded by specialized personnel. The safety of thrombolytic therapy is mainly evaluated by the incidence of intracranial hemorrhage. During thrombolysis, if there is headache, vomiting, and other suspected intracranial hemorrhage or other parts of the bleeding, stop thrombolysis immediately. At the same time, review the head CT. If there is no symptom of appeal, the head CT is reviewed 24 hours after thrombolysis. The significant deterioration of nerve function caused by intracranial hemorrhage (increased NIHSS score ≥4 points) will be considered as symptomatic intracranial hemorrhage.

#### 2.6.2 Evaluation of efficacy and prognosis

A trained neurologist who will be unaware of the patient's morbidity was evaluated by the National Institutes of Health Stroke Scale (NIHSS score) at the time of patient admission, 24 hours after treatment, and 7 days after treatment. After discharge, the patient's prognosis was evaluated by the modified RanKin scale (mRS). We defined mRS score 0 to 1 points as cured and 0 to 2 points as good prognosis. After 7 days of treatment, the NIHSS score is reduced by 4 points and it is considered that it will be judged as effective in the early stage, and the NIHSS score is increased by 4 points or death will be judged as invalid or worse (Table 1).

**Table 1:**
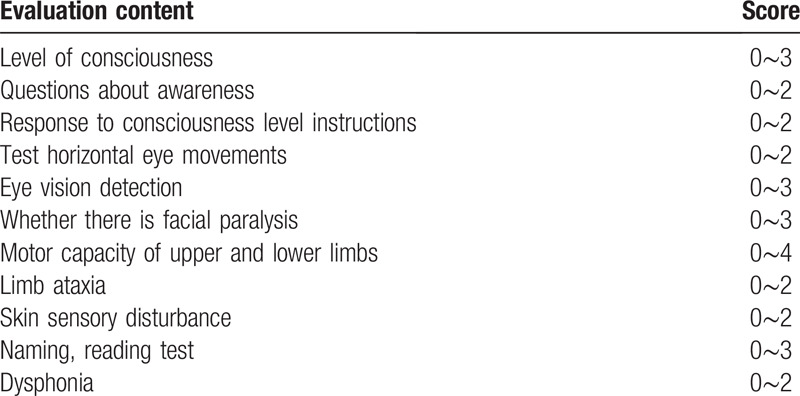
National Institutes of Health Stroke Scale.

### 2.7 Randomization and blinding

1. The concealment of random schemes: After the scheme design is completed, the random schemes are packed into sealed, light-tight envelopes encoded in sequence; then they are handed over to the person in charge of the project, and the qualified cases are selected before opening the envelope to determine case grouping.
2. The recorder fills in the case report form with basic data and observation of local and systemic symptoms of the patient.
3. We will make all kinds of documents in a unified way
4. Check and confirm the correctness and completeness of all research data records and reports and case report forms, and ensure consistency with the original data.

### 2.8 Statistical analysis

All data will be analyzed using statistical software SPSS 25.0 (SPSS Inc, Chicago, IL). Under the condition that the measurement data meet the normality and homogeneity of variance, the before and after data of the same group are compared using the paired *t* test. If the data in the group are not normal, the Wilcoxon signed rank sum test in the nonparametric test can be used. The measurement data meet the conditions of normality and homogeneity of variance, and the data of the same period between the 2 groups are compared using the independent *t* test method. If the 2 sets of data do not meet the normal distribution, the Mann-Whitney *U* test in the nonparametric test makes it easy to compare the average level of data with no statistical significance. The count data are expressed in terms of frequency (f) and composition ratio. The composition ratio or percentage is tested by *χ*^2^ test. If the theoretical frequency T < 5 but T ≥1 in the data, the *χ*^2^ test with continuity correction is used. If any theoretical number is 0, Fisher test is used. To evaluate the treatment effect, Wilcoxon signed rank sum test will be used. All tests used a 2-sided test, with *P* < .05 indicating that the difference will be statistically significant.

### 2.9 Data management

**Figure 2:**
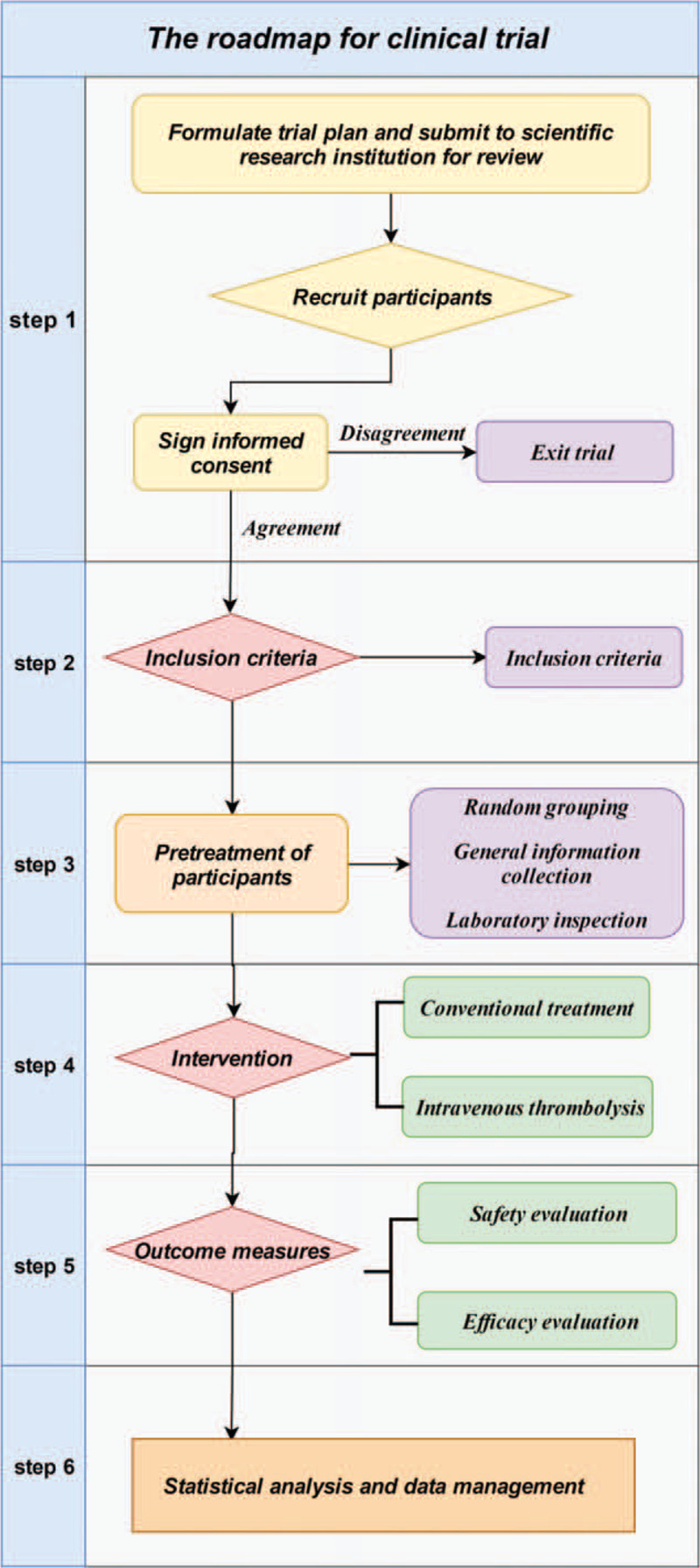
Study design flow chart.

The information obtained from each participant's evaluation will be recorded and printed on paper. Then, this information will be handwritten on the paper document case report form, which is used in the EXCEL file for future statistical analysis. According to the Personal Information Protection Act, we will not disclose the names of all participants and the unique identification numbers provided during this period to determine the participants. All participants will be informed that the clinical data obtained during the trial will be stored in the computer. Participants’ written consent will be stored by the lead investigator (Fig. 2).

## 3 Discussion

Intravenous thrombolytic therapy for ischemic stroke in the ultra-early stage has been proved to be an effective treatment in most randomized trials. However, ultra-early intravenous thrombolysis has a strict treatment time window (onset time <4.5 hours). Exceeding this treatment time window will increase the incidence of symptomatic intracranial hemorrhage, and severe cases may be life-threatening. WUS starts in sleep and is a more common form of ischemic stroke. Its incidence rate can be as high as 25%.^[17]^ Since patients or witnesses cannot provide accurate time of onset, the start time of stroke is still “normal at last.” Therefore, even if the patient or eyewitness finds out, the prescribed time window has already been exceeded. However, some recent research results prove that the original method does not seem appropriate. The study by Kim et al^[18]^ also focused on patients with WUS and stroke patients with unknown onset times during the day without witnesses. He found that nearly half of WUS patients will be immediately awake as soon as clinical symptoms appeared. Roveri et al^[19]^ divided the study subjects into 2 groups, the WUS group and the intravenous thrombolysis group within 3 hours of onset. Early CT manifestations will be applied to the early CT score of the Alberta Stroke Project (ASPECTS), and it was found that regardless of the risk factors and stroke severity Or the ASPECTS score is very close between the 2 groups. There was no statistically significant difference in the early CT scan ASPECTS scores between the 2 groups, indicating that the ischemic event of WUS patients occurred shortly before awakening or awakened as soon as the disease occurred. The author also pointed out that the prognosis of patients in the intravenous thrombolysis group was significantly better than that in the WUS group. The most representative epidemiological evaluation of WUS comes from a 2-center study of 1853 patients with ischemic stroke, including 273 WUS patients. The results show that compared with non-WUS patients, the incidence of WUS patients older age and higher NIHSS score on admission. However, if the time window is not considered, 35% of the patients are suitable for thrombolytic therapy. Then, if we can more accurately assess the onset time of WUS patients, a large number of patients will benefit from thrombolytic therapy. The study found that the mismatch of DWI/FLAIR has a high specificity and sensitivity for the judgment of patients within 3 to 4.5 hours. Thomalla et al^[20]^ observed 543 patients within 4.5 hours of onset. Under the field strength of 1.5 T, the specificity of DWI/FLAIR mismatch judgment of stroke patients within 4.5 hours of onset was as high as 78%. Morelli et al^[21]^ collected 27 WUS patients and 143 non-WUS patients and received intravenous thrombolysis. The results showed that the proportion of patients with mRS ≤1 point was 36.4%. The author mentioned in the conclusion that the selected WUS patients are suitable for intravenous thrombolysis. This study is based on the previous study, and has a purposeful choice for WUS patients who are given thrombolytic therapy. The imaging findings are consistent with DWI/FLAIR mismatch. In summary, we speculate that the selected WUS patients are safer to use intravenous thrombolytic therapy, which is expected to improve the patients’ early efficacy and long-term prognosis. However, whether the statistically significant benefits can be obtained remains to be observed by expanding the sample size. There is no consistent conclusion about the pathogenesis of WUS. WUS is more likely to manifest as small vessel disease. Male patients with sleep apnea are more likely to have WUS, and frequent snoring may be one of the mechanisms of WUS pathogenesis.^[22,23]^ The incidence of WUS patients is relatively high, and the effectiveness and safety of intravenous thrombolysis under the guidance of multimodality imaging treatment of WUS need to be further explored by prospective, large-scale studies.

## Acknowledgments

The authors thank all patients who participated in this study. The authors express their heartfelt thanks for the support given by the Science and Technology Department of Hebei Province.

## Author contributions

**Conceptualization:** CYZ.

**Formal analysis:** CLW and BY.

**Investigation:** DML and QYS.

**Supervision:** CYZ and YLL.

**Writing – original draft:** CYZ.

**Writing – review & editing:** CYZ and HL.
